# Toward More Robust Hand Gesture Recognition on EIT Data

**DOI:** 10.3389/fnbot.2021.659311

**Published:** 2021-08-11

**Authors:** David P. Leins, Christian Gibas, Rainer Brück, Robert Haschke

**Affiliations:** ^1^Research Institute Cognitive Interaction Technology, Bielefeld University, Bielefeld, Germany; ^2^Medical Informatics and Microsytems Engineering, Faculty of Life Sciences, University of Siegen, Siegen, Germany

**Keywords:** electrical impedance tomography, gesture recognition, artificial intelligence, neural networks, deep learning, data analysis

## Abstract

Striving for more robust and natural control of multi-fingered hand prostheses, we are studying electrical impedance tomography (EIT) as a method to monitor residual muscle activations. Previous work has shown promising results for hand gesture recognition, but also lacks generalization across multiple sessions and users. Thus, the present paper aims for a detailed analysis of an existing EIT dataset acquired with a 16-electrode wrist band as a prerequisite for further improvements of machine learning results on this type of signal. The performed t-SNE analysis confirms a much stronger inter-session and inter-user variance compared to the expected in-class variance. Additionally, we observe a strong drift of signals within a session. To handle these challenging problems, we propose new machine learning architectures based on deep learning, which allow to separate undesired from desired variation and thus significantly improve the classification accuracy. With these new architectures we increased cross-session classification accuracy on 12 gestures from 19.55 to 30.45%. Based on a fundamental data analysis we developed three calibration methods and thus were able to further increase cross-session classification accuracy to 39.01, 55.37, and 56.34%, respectively.

## 1. Introduction

In the past decades, we have seen tremendous progress in the development of bionic hands and other prosthetic devices providing multiple, self-powered degrees of freedom to restore lost dexterity for upper-limb amputees. One, maybe the most advanced example nowadays, is the Luke Arm^1^, which is the commercial version of the Modular Prosthetic Limb (MPL) providing up to 26 articulating degrees of freedom (DOF) via 17 actuators from shoulder to hand and sensory feedback via vibrotactile sensors (Perry et al., 2018). Traditionally, electric prosthetic devices are controlled via surface electromyography (sEMG), where the electrical activity of surface muscles is recorded from electrodes attached to the skin (Farina et al., 2014). However, these electrodes are only passive sensors, which amplify the body's own electrical activity. Thus, the signal quality of their measurements is very limited. Another, non-invasive approach that promised to overcome this limitation was tactile myography: a high-resolution array of tactile force sensors, worn as a bracelet around the forearm, is measuring the bulging of muscles with up to 320 tactile cells (Kõiva et al., 2015). Using simple linear regression methods, Connan et al. (2020) achieved a remarkable success rate of 70% in continuous hand pose control trained on a few singular hand poses only. However, a limitation common to both of these approaches is their restriction to the surface of the skin. To also reach deeper muscular layers, electrical impedance tomography (EIT) has emerged in the past few years as a potential new alternative for myographic signal acquisition. EIT, proposed by Henderson and Webster (1978), uses pair-wise impedance measurements from surface electrodes surrounding an object to recover the impedance distribution of its inner structure, which in turn allows conclusions to be drawn about the internal structure of the object itself. Originally, EIT has been used successfully in clinical applications imaging the thorax region of the human body (Khan and Ling, 2019). Zhang and Harrison (2015) first reported its use for hand gesture recognition employing a mobile eight-electrode system. Using a support vector machine (SVM) with a standard polynomial kernel, they achieved an in-session classification accuracy of up to 97%, distinguishing eight gross gestures. However, when considering reproducibility between sessions, i.e., after removing and re-attaching the sensor band, the accuracy dropped by a range of 14–29%. Considering universality, i.e., the generalization capabilities between different users, the accuracy again dropped significantly to a level around 40%. Later results from Wu et al. (2018a) confirm these poor generalization capabilities.

To better understand the underlying changes in the data between different sessions and users, the present paper aims for an in-depth data analysis using modern visualization techniques. This will pave the way toward more realistic applications requiring high recognition accuracies also across sessions. Retraining the system before each usage is laborious and simply not practical. Our analysis reveals that inter-class variance of the data is much smaller than cross-session and cross-user variances, which both range at a similar level. We also observe a significant data drift within sessions, which calls for a continuous co-adaptation scheme as suggested, e.g., by Beckerle et al. (2018), to maintain high-quality control. Based on our analysis, we propose different normalization techniques and modern deep neural network approaches for machine learning, which considerably improve generalization capabilities.

## 2. State of the Art

### 2.1. Electrical Impedance Tomography (EIT) for Hand Gesture Recognition

EIT is a non-invasive imaging technology, to recover the inner impedance distribution (and thus the inner structure) of conductive objects. In the medical domain, it is predominantly applied to imaging of the thorax region, e.g., the lung (Khan and Ling, 2019). To this end, a low-magnitude, alternating electrical field is applied to the body via surface electrodes. This field is unequally distorted by different layers of tissue due to their varying impedance (caused by different ion concentrations in the tissue) and the resulting potential distribution is measured with another set of electrodes on the body surface. Based on these measurements the interior impedance distribution can be determined using various reconstruction techniques, see Holder (2015) for a detailed description of the methodology.

**Figure 1:**
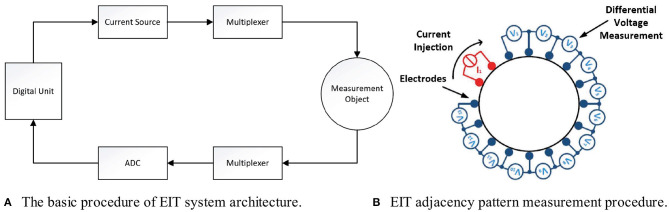
General structure of an EIT system: In **(A)** the overall system structure is shown, where a central unit controls the whole system and processes measured values. In **(B)** the adjacency measurement protocol for the first cycle is shown, where red indicates electrodes used for current injection and blue indicates electrodes used for voltage measurements.

The overall approach is illustrated in Figure 1A: A current source generates an analog alternating signal with a frequency in the beta range (10 kHz–1 MHz). These frequencies have proven to be best suited for the measurement of tissue impedance. To avoid damage to the tissue, the net current injected should be zero. Thus, according to the standard (DIN/EN 60601-1 VDE 0750-1:2013-12, 2013), the DC component needs to be smaller than 10 μA, which is achieved by suitable filtering. A multiplexer distributes the voltage to the injecting electrodes on the object's surface. The signal from the measurement electrodes is multiplexed back to an analog-digital converter (ADC) and then fed into the central processing unit that computes the amplitude and phase shift relative to the injected signal. To this end, several oscillations of the signal are recorded and subsequently processed.

The EIT systems for hand gesture recognition published so far comprise 8–32 electrodes arranged circularly around the forearm as illustrated in Figure 1B for a system of *N* = 16 electrodes. The electrodes are aligned and positioned equidistantly to achieve optimal coverage of the arm's circumference. Most current work applies a four-electrode measurement method approach, where the current is injected via two electrodes and the potential distribution is measured via two other, distinct electrodes. This avoids erroneous measurements compared to the two-electrode measurement, which injects and measures via the same pair of electrodes.

Obviously, there exists a multitude of electrode combinations that could be used for injection and measurements. The most common approach used in literature is the adjacency pattern shown in Figure 1B: Two adjacent electrodes are used for current injection (red-colored) and two other adjacent electrodes are subsequently used for voltage measurements (blue-colored), resulting in *N* − 3 differential voltage measurements *V*_1_-*V*_13_, called a *cycle*. For the next cycle, the arrangement is rotated by 360°/*N*, such that the current is injected via the next pair of adjacent electrodes. After a full rotation, *N* cycles and thus *N*(*N* − 3) measurements were gathered, which form a *frame*. According to Kaufmann (2015), this adjacency measurement pattern provides higher data density and thus more information content than other patterns.

The existing EIT systems developed for hand gesture recognition differ by the number of electrodes used, the details of the current generation and readout circuits, as well as their injection and measurement scheme. The first EIT system, introduced by Zhang and Harrison (2015) used a ring of *N* = 8 electrodes with a two-electrode measurement for all *N*(*N* − 1)/2 = 28 combinations of electrode pairs. In their successor system they compared rings of 8, 16, and 32 electrodes in a modular design (Zhang et al., 2016) that allowed for both, two- and four-electrode measurements as well as different measurement patterns. To handle the much larger number of electrode pairs, they reduced the sensing time by a factor of 10 employing customized hardware. In both setups, they used a 40 kHz excitation signal and recorded 250 voltage samples for each measurement (i.e., ca. 5 periods of the injected signal), whose overall power was subsequently computed with a discrete Fourier transformation, then serving as a scalar measurement value.

Wu et al. (2018b) developed an EIT system to control a hand prosthesis. They used eight electrodes and the adjacency pattern as well. Jiang et al. (2020) compared two different wristband layouts, each comprising 16 electrodes in total. Additionally to the traditional layout with all electrodes arranged in a single ring, they also considered a layout of two separate rings comprising eight electrodes each. This layout proved to be slightly superior as it can measure the muscle tension at two different locations.

Yao et al. (2020) compared gesture recognition rates of different electrode materials and shapes, using a portable 8-electrode EIT device. They tested rectangular copper, curved copper, conductive cloth, and (circular) medical electrodes. While the first three were fixed using elastic bandages, the medical electrodes were fixed using medical tape and additionally conductive fluid was applied. The secondary aim of the comparison was to investigate the influence of electrode-skin contact impedance on gesture recognition rates. Yao et al. concluded that a higher contact impedance contributes to a higher recognition rate, and found quadratic copper electrodes to achieve the best results.

**Figure 2:**
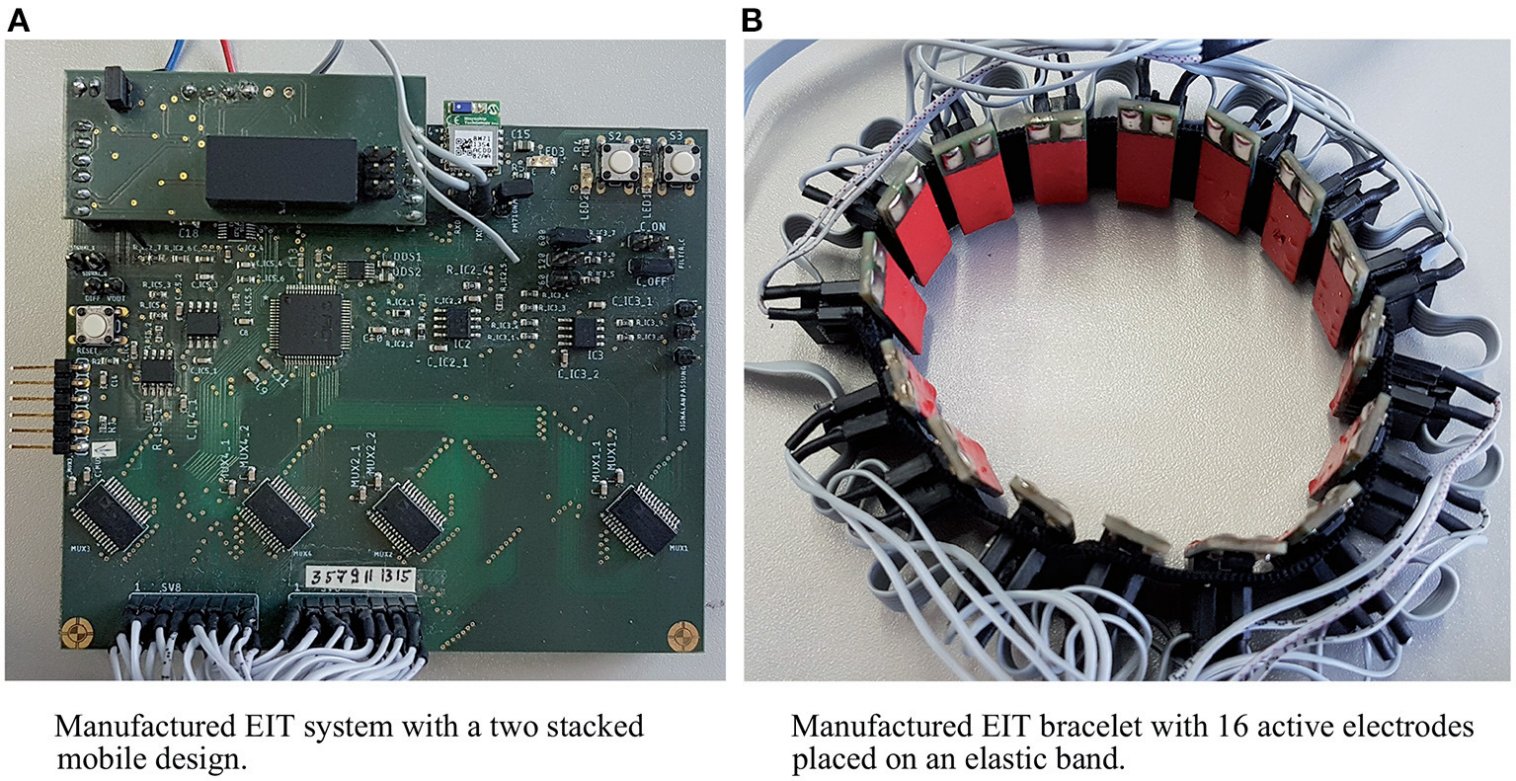
EIT system used to record the data analyzed in this work: The central control unit **(A)** controls the current injection and voltage measurement. The electrode ring with 16 electrodes **(B)** exhibiting protective pads (red) and two silver pads indicating separate electrodes for current injection and measurement.

Finally, the system developed by Gibas et al. (2020) that was used to acquire the dataset analyzed in the present paper, used a wristband of 16 rectangular copper electrodes as well. The design is modular to allow for different measurement patterns, excitation signals, and data acquisition schemes. As shown in Figure 2, separate electrode contacts for injection and measurement were used. In the active electrode, the measuring signals are pre-amplified by a factor of two via the multiplexer architecture to a differential amplifier. In all experiments considered in this paper, the excitation frequency was fixed to 50 kHz (sinusoidal waveform, rms value of 5 mA) and the adjacency pattern was used for data acquisition resulting in *N*(*N* − 3) = 208 measurements per frame. 50 kHz were used in this study, because higher frequencies rapidly increase the complexity of the electrical layout, whereas lower frequencies reduce the frame rate. The hardware integrated filters also have been optimized for 50 kHz. Similarly to Zhang et al. (2016), five periods of the excitation/measurement signal were recorded to determine the signal amplitude via peak-to-peak analysis, which serves as a single measurement. Phase information was neglected for now, as in most other previous work as well. The employed ADCs have a resolution of 16 bits (at least 12 bits are required for EIT) and a sampling rate of 2 MSPS. Thus, we can sample 40 values per period of the 50 kHz excitation signal, which corresponds to a twentyfold oversampling according to the Nyquist-Shannon sampling theorem. Overall, the system provides a data frequency of 1.2 frames per second.

### 2.2. Machine Learning Approaches for EIT-Based Gesture Recognition

Existing machine learning approaches to classify hand gestures based on EIT signals can be divided into two classes: those which first apply classical image reconstruction methods and those which operate directly on the raw data. Various machine learning approaches have been used, ranging from simple nearest-neighbor classifiers, over support-vector machines (SVM) to modern deep neural networks (DNN). As all previous work has used a different set (and also a different number) of gestures to distinguish, their results are only hardly comparable. The more gestures need to be distinguished and the more similar those gestures are, the more difficult the classification task becomes.

In Wu et al. (2018b), three distinctly trained neural networks were used to recognize a total of 11 distinct gestures divided into three hierarchical groups of 5, 5, and 3 gestures (the gesture “fist” was present in all groups), where each network was responsible for a single group. In a study involving five volunteers, the neural networks achieved accuracies of 98.5% (3 gestures), 92 and 97%, respectively using cross-validation on data recorded in a single session. Further experiments showed that training a new model was necessary when a user starts to wear the device, i.e., the system lacks inter-session generalization. Using the combined data of several sessions across five subjects, a comparison between several variants of k-Nearest-Neighbor and support-vector machines, as well as two neural networks with 100 and 10 hidden neurons, respectively was performed. This comparison demonstrated that the neural networks did not only reach competitive scores, but also required significantly less time to train.

Zhang et al. (2016) performed classical image reconstruction employing the EIDORS toolkit (Adler and Lionheart, 2006), before they performed SVM-classification on the reconstructed 16 × 16 pixel images. In a trial with 10 participants, the accuracy on a set of 11 gestures was evaluated with 8, 16, and 32 electrode measurements and two-pole as well as four-pole measurement schemes. The results showed that four-pole sensing was superior in all setups—with accuracies increasing with the number of electrodes available. Using 16 and 32 electrodes, they achieved an in-session accuracy of 92.4 and 94.3% at frame rates of 16 and 3 Hz, respectively. Thus, they concluded that for interactive applications a 16-electrode setup would be more beneficial given the limited frame rate of the 32-electrode setup, which is only slightly more accurate.

Yao et al. (2020) tested six subjects performing three distinct hand gestures with the device attached on the wrist. For each electrode setup, 10 measurements per gesture were recorded without taking the device off. Four-pole sensing was used and measured electrode-skin contact impedances were used as additional input features. As classification algorithms a radial basis function (RBF) kernel SVM, a bagged tree ensemble, and a quadratic discriminant classifier were applied. They found the RBF SVM to be the best performing classifier with quadratic copper electrodes, resulting in an average (in-session) classification rate of 95% across all subjects.

Using a set of eight gestures, Jiang et al. (2020) compared various classification methods [decision tree, SVM, and neural network (NN) with 10 hidden neurons] with respect to the two wristband layouts. The results showed that the split wristbands, placed at a distance of 10 cm, could better distinguish similar gestures. The single band with 16 electrodes achieved accuracies of 97.9% (decision tree), 97.5% (SVM), and 95.4% (NN), while the double band layout achieved almost perfect classification with 99.5, 99.4, and 99.5% accuracy, respectively. Note that in this case, for model training a five-fold cross-validation approach was chosen on the whole dataset, such that the trained models have seen each data point at least once.

In the field of EIT image reconstruction, neural networks have often been applied to solve the forward and inverse problems (see Khan and Ling, 2019 for a review) and recently also more specialized deep learning architectures are being explored in that domain (Kłosowski and Rymarczyk, 2017; Hamilton and Hauptmann, 2018; Wei et al., 2019). In contrast to that, classical machine learning methods have been predominantly applied for gesture recognition so far. Only the works of Gibas et al. (2020), which is the basis for the present paper, and the works of Wu et al. (2018a,b) and Jiang et al. (2020) have considered neural networks and only rather classical MLP variants. In the present work, we compare some more specialized network architectures, particularly convolutional neural networks (CNN) and two-input networks to achieve better cross-session generalization.

## 3. Data Description

**Figure 3:**
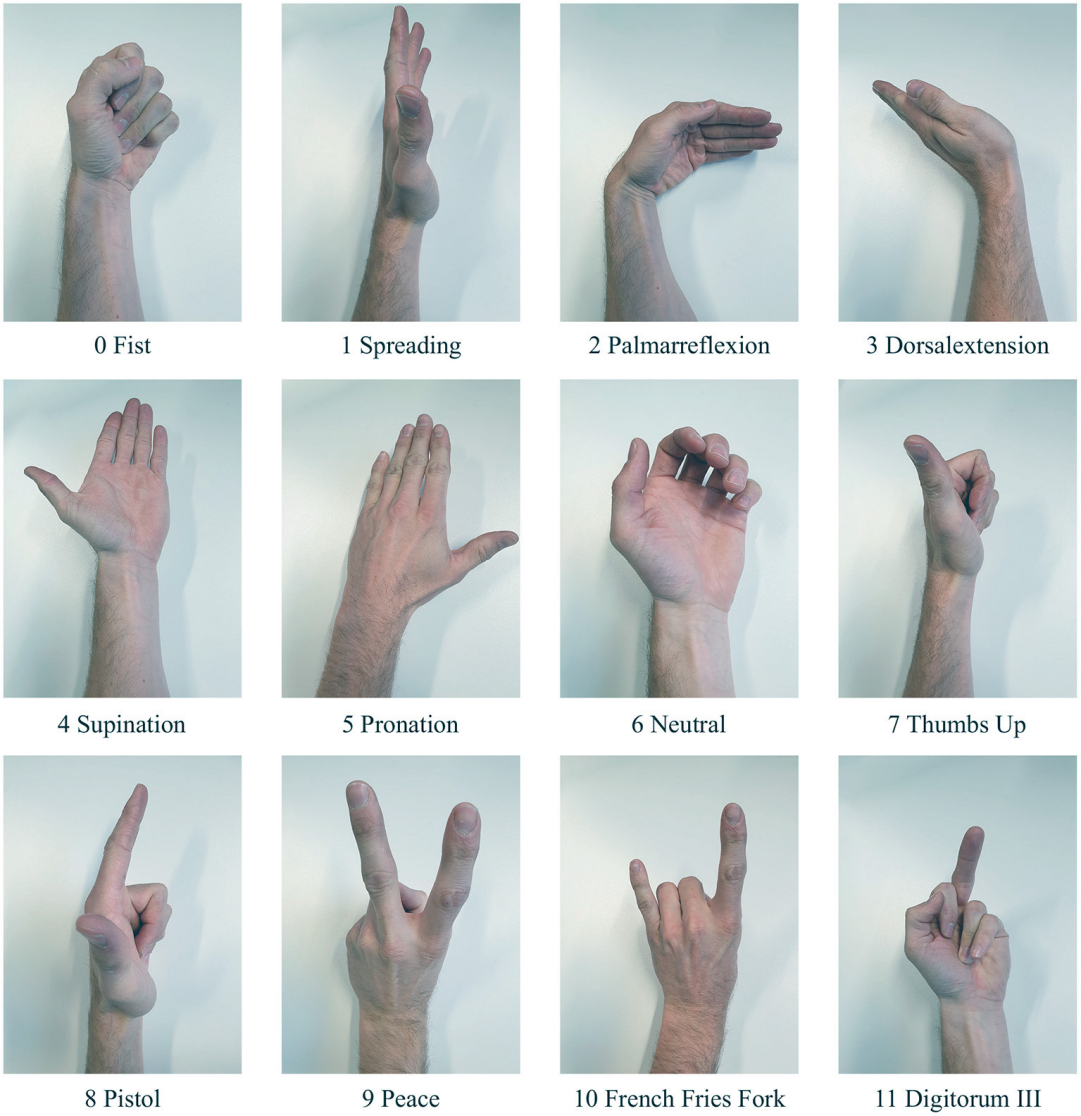
The used gesture set for the experimental setup of the study.

In all experiments considered in this work, the left arm was taken as the measurement object. It is assumed that measurements on the right arm yield symmetrically correlated results. To ensure that only required muscle groups get activated for a specific hand gesture, subjects are asked to rest their elbow on a table, letting the forearm point vertically upwards. If both, flexors and extensors are relaxed, a relaxed claw hand is resulting, which serves as a neutral reference gesture for calibration in section 4.5. The remaining gestures shown in Figure 3 cover full closing and opening of the hand (0, 1), wrist joint motions (2–5), and various finger gestures (7–11). The gestures are shown in Figure 3 along with their labels, which are used for later referencing.

To record a single gesture, the participant was asked to mimic a gesture displayed on the screen and keep it as still as possible. During that time, we recorded 10 consecutive frames at a rate of 1.2 FPS as described in section 2.1. After recording these 10 frames, the participant was prompted to mimic a new gesture. No data was recorded for the transitions between gestures. This procedure was repeated until all 12 gestures were conducted by the participant in series, concluding a single *iteration*. A recording *session* typically comprises several of those iterations, ranging from 5 to 8 across different sessions in our experiments. Note that between iterations the device was not taken off or moved. However, a *new* session was started as soon as the EIT device was (re)attached to a subject's arm. We reference sessions by an ID composed of a unique alphabetic subject identifier and a subject-unique session number, e.g., *A1* for session 1 of subject *A*. Sessions E2 and E8 have eight iterations, A1, E1, E3, E5, and E7 have seven iterations, B1, C1, and D1 have six iterations, and E4 and E6 have five iterations.

To reduce inter-session variance, we took care to place the EIT device with its electrodes in the same position relative to the subjects' arms when starting a new session or switching subjects. While this reduces inter-session variance due to a rotation of the wristband, there are other sources of variance that are not easily controllable, namely: salt formation on the skin, indirect electrode-skin contact, or general pressure fluctuations of the electrodes. These external contributions can affect measured impedances significantly. Salt, already resulting from light sweating, acts as an electrically promoting medium that can massively influence the actual contact impedance between the EIT system and the measured object. If an irregular spread of salt on the electrodes is assumed, a proportionate inaccuracy in measurements must be expected.

Furthermore, electrode-skin contact can generally suffer from various other uncontrollable parameters. For example, a hair between an electrode and the skin will form an additional barrier posing an additional source of error, since a supposedly higher impedance is detected at the affected electrode. The elastic band used here ensures that the contact between the electrodes remains as constant as possible, but an electrode on the ulna or salivary bone, for example, has a significantly higher contact pressure than an electrode placed in a gap. Finally, longer use of the EIT system will warm up the device and its electrodes due to the flow of current and the contact to the skin. This will result in an increased conductivity (or reduced resistance) between the electrodes and the skin, which can be noticed as in-session drift in the data.

Upon inspection of the existing dataset from Gibas et al. (2020), we remark that in iteration 1 of session E1 no samples of gesture 3 are available, and in iteration 7 of session E8 samples of classes 9, 10, and 11 are missing. Other than that, we did not observe any outliers in the data; thus we conclude that the technical acquisition process worked fine during the course of the experiments.

### 3.1. Additionally Recorded Data

As an initial analysis of the dataset revealed different sessions of the same user to be quite dissimilar, we conducted a second measurement experiment focused on increased within-user data variance. To this end, 20 sessions were gathered from a single subject (later referenced as subject *F*). In contrast to the previous experiment procedure, these recordings were limited to a *single* iteration per session. However, all sessions were recorded in a row. The data set, including the additionally recorded data, is available as open access publication (Leins et al., 2020).

### 3.2. Pre-processing

A major strength of (deep) neural networks over classical machine learning approaches is that features are automatically learned. Thus, our models operate on the raw input data, which is just normalized to the range 0 to 1.

## 4. Methods

### 4.1. Classification Tasks

Considering the variance in the combined dataset we are able to study EIT-data-based recognition of 12 hand gestures under three different aspects: within-session, across-session, and across-subject. Intuitively within-session gesture recognition poses the easiest problem, as subject physiology and device placement remain constant. Cross-session recognition is more difficult, as a slight mismatch in device placement not only results in higher data variance because of a rotational or translational offset, but also influences the contact impedances of the electrodes directly as discussed in the previous section. Finally, cross-subject recognition additionally introduces changed subject physiology, which requires a higher level of abstraction.

As mentioned in section 2.2, previous work primarily focused on in-session analysis and reported accuracies above 90%. On the other hand, considering cross-session and cross-user experiments the accuracies dropped to the point where the results are no longer suitable for application.

With our EIT dataset we attempted to verify these findings as follows:

1. We evaluated a set of distinct classifiers with a stratified random shuffle split (75/25% for each class individually), to verify that within-session classification can be considered trivial.
2. Following the within-session evaluation procedure of Zhang and Harrison (2015), we evaluated the same classifiers as before on within-session data with a leave-one-iteration-out train/test split, meaning a single (randomly chosen) iteration was used as the test set while the remaining iterations were used for training. This experiment will reveal if generalization on an unseen iteration is possible.
3. Further, we analyzed cross-session recognition quality with the two participants in our set for which more than a single session is available. In this case, train/test splits were performed on a leave-one-session-out basis for each combination of sessions.
4. Finally, we evaluated cross-user accuracy by analyzing the prediction quality of leave-one-subject-out train/test splits for each combination.

As baseline models, we chose a variation of SVMs with distinct kernels and four multilayer perceptron (MLP) neural network architectures. As SVM kernels we chose a linear kernel, an RBF kernel, and three polynomial variants with 2, 3, and 6 degrees of freedom. Besides the choice of kernel and degree, the default scikit-learn parameters were used. The first MLP (*MLP 1*) is the same architecture that performed best in Gibas et al. (2020): it has two hidden layers with 128 neurons each, uses ReLU activations, and has a dropout rate of 50% after both layers. The second MLP (*MLP 2*) has three hidden layers with 256, 128, and 64 neurons respectively with ReLU activations and a dropout rate of 50% after the first two layers. To reveal the impact of the dropout regularization, both MLPs were also tested without dropout (*MLP 1 Unreg* and *MLP 2 Unreg*). For each model, we use categorical cross-entropy loss and the Adam optimizer (Kingma and Ba, 2014).

### 4.2. Convolutional Neural Networks (CNN)

Considering the circular structure of the measuring process, a frame of 208 measurements can be reshaped into a 16 × 16 × 1 tensor explicitly preserving the structure of 16 cycles composing a frame. With the expectation that harnessing this structural information with convolutional layers would improve model performance, we also explored some basic convolutional neural network (CNN) architectures. These networks consist of convolution blocks, comprising a 3 × 3-kernel convolution, followed by batch normalization and ReLU activation. After a certain number of convolution blocks, the output is eventually flattened and fed into a fully-connected classification layer. The inputs to all convolution layers are zero-padded to maintain the input dimensions. We provide results for our two best-performing CNNs, denoted as *CNN 1* and *CNN 2*. *CNN 1* uses four convolution blocks with 64 filters, each followed by a dropout layer with 25% dropout rate. All convolution blocks except the first one, use a dilation rate of 3 to increase the receptive field while maintaining the number of kernel parameters. *CNN 2* uses only three convolution blocks with 64 filters and no dropout. Only the last block uses a dilated convolution with a rate equal to two.

### 4.3. Raw Data Analysis

To gain deeper insight into the spatial characteristics of our data set, and particularly to understand inter-class, inter-session, and inter-user variability we performed a t-SNE analysis on the overall data set. t-SNE or t-distributed stochastic neighbor embedding (Maaten and Hinton, 2008) is a visualization method aiming for a non-linear projection of the data onto a low-dimensional (typically two-dimensional) space preserving the distribution of distances observed in the original, high-dimensional data space. The perplexity parameter of t-SNE weighs the influence of neighboring points in the distribution based on their distance. A lower perplexity focuses on the local structure, whereas a higher perplexity shifts the focus toward the global structure. The cost function of t-SNE, i.e., the Kullback-Leibler divergence between the joint probabilities in the embedding space and the high-dimensional data, is not convex and optimized via gradient descent. Hence, additionally to perplexity, the results depend on learning rate, computation steps, and initialization.

Note that in t-SNE embeddings, the (relative) size of clusters and their rotation are irrelevant. Also, distances between clusters in t-SNE projections are not necessarily meaningful, because the algorithm retains probabilities of distances rather than the distances themselves. Further, data distances after projection are heavily influenced by the chosen perplexity and the overall data density.

In our experiments we use scikit-learn's t-SNE implementation, always setting the random seed to 42 and the iterations (computation steps) to 5,000. A perplexity of 90 was chosen for class/session projection, while for the iteration-focused projection it was set to 100.

### 4.4. Iteration Drift Experiment

As the t-SNE analysis revealed the presence of in-session drift, we performed a series of experiments to further analyze and quantify this drift between iterations. To this end, we trained individual classifiers on the data of a single iteration and evaluated them on the data of the subsequent iteration. When there is little to no drift, the classifier should successfully generalize across these two iterations. If the datasets of the two iterations differ more strongly, e.g., due to drift, we would expect a worse generalization capability. To gain statistical relevance, the results from five independent, randomly initialized network trainings were averaged for each configuration. This analysis was performed for the datasets of all subjects individually. Subject *F* was excluded, because we only have a single iteration per session for this subject.

### 4.5. Manual Calibrations

In case of data drift, it is common practice to employ difference measurements to an initial reference measurement to focus the analysis/classification on the relevant changes *relative* to this reference. This idea also applies to EIT for gesture recognition: As we are only interested in relative changes of muscle activity rather than the subject's (absolute) physiology, we expect an improvement in classification accuracy when using difference measurements. As long as the regions of muscle activation remain consistent across iterations, sessions, or subjects the difference to an individual reference posture should provide a more robust basis for classification of individual gestures. Between sessions of the same subject, a calibration based on such a reference measurement should also cancel out stationary offsets in measurements caused by a changed conductivity of the electrodes.

The *neutral* gesture (no. 6) is the most suitable to use as a reference measure because we expect the relaxed state of arm and hand to be more similar between subjects than other gestures. Given that we recorded 10 consecutive measurement frames for each gesture within a single iteration, we have several options to compute a reference measurement: Firstly, we could compute the mean of these 10 measurement frames within the *first* iteration only. This approach, denoted as *global* calibration, will shift the data of all iterations within a session by the same amount. This might allow compensating variations across subjects and sessions but cannot contribute to a normalization of drift within a session. To handle this as well, secondly, we consider employing a different reference measurement for each iteration by averaging across the 10 neutral-gesture measurements within the respective iteration. This approach is denoted as *local* calibration as each iteration is normalized on its own. While the former approach will be more suitable for real-world applications, as calibration is necessary only at the beginning of a session, the latter can be used to normalize the data set with significantly reduced drift effects for further analyses.

To verify and quantify the anticipated improvements by the proposed calibrations, the raw data classification experiments were repeated with both calibration variants and compared to each other.

### 4.6. Learned Calibration

As we discuss in section 5.4, the naive approach to calibration, simply subtracting the reference measurement from an input measurement, does not yet yield satisfying results. Alternatively, we considered a learned-calibration approach, where the neural network was fed with two inputs, namely the actual input measurement to classify and the reference measurement (employing *local* calibration). This allows the network to learn its own, non-linear way to offset the measurement with the reference gesture. Within this scope, we considered two approaches to dual input processing: firstly, concatenating both samples directly after the input layer, i.e., considering a monolithic, 512-dimensional input for MLPs or a 16 × 16 × 2 tensor for CNNs, and secondly, processing both inputs separately in structurally identical, but individually trained computation branches before concatenating the output of both branches and processing them together in some subsequent, common layers. While the first approach attempts *early* fusion (and is denoted with the suffix *E*), the latter approach (denoted with the suffix *L*) allows for some non-linear pre-processing before attempting *late* fusion exploiting a presumably better linearized embedding space.

In this paper we report results for the following set of learned-calibration MLP networks:

- **MLP 3** processes the input with three hidden 128-neuron layers with ReLU activations, followed by the classification layer (monolithic input case). In the dual input case, the first two layers are duplicated for the secondary output. After traversing two layers each, the outputs are concatenated before traversing the last hidden, and the classification layer.
- **MLP 4** with monolithic input is exactly the same architecture as *MLP 2*. For two inputs, the respective samples are processed separately in two identical copies of the first three layers (256, 128, and 64 neurons with ReLU), after which the outputs are concatenated and fed to the classification layer.

**Figure 4:**
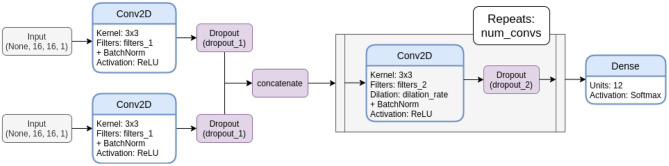
Template network architecture for dual input CNNs. Note that this depiction shows the late fusion variant. In the early fusion variants, the inputs are concatenated directly after the input layers and processed in one branch, instead of providing an individual convolution block for both inputs before fusion.

For CNNs, we considered a whole bunch of architectures differing in the number of computed filters, the number of convolutional layers, the employed dilation rate, and the dropout rate used for regularization. Figure 4 displays the template architecture that was employed to derive all dual-input CNNs from and Table 1 lists all considered CNN architectures with their parameters. With this explorative approach, we empirically scanned the hyper-parameter space for the most promising architectures. Note that the two parallel computation branches of the late fusion models share their architectures, but are trained independently, i.e., they do not use weight sharing.

**Table 1:** Parameters of dual-input CNN models derived from the template architecture shown in Figure 4.

	**Filters_1**	**Filters_2**	**Dropout_1**	**Dropout_2**	**Num_convs**	**Dilation_rate**
CNN 3 L	64	64	0.0	0.0	3	3
CNN 3 L D-25	64	64	0.25	0.25	3	3
CNN 3 L D-50	64	64	0.50	0.50	3	3
CNN 4 L	64	64	0.0	0.0	1	2
CNN 5 L	32	32	0.0	0.0	3	3
CNN 6 L	64	64	0.0	0.0	1	1
CNN 6 L D-25	64	64	0.25	0.25	1	1
CNN 6 L D-50	64	64	0.50	0.50	1	1
CNN 6 E	64	64	0.0	0.0	1	3
CNN 6 E D-25	64	64	0.25	0.25	1	3
CNN 6 E D-50	64	64	0.50	0.50	1	3
CNN 7 L	64	64	0.0	0.0	2	2
CNN 8 E	64	64	0.0	0.0	1	2
CNN 8 E D-25	64	64	0.25	0.25	1	2
CNN 8 E D-50	64	64	0.50	0.50	1	2
CNN 9 E	128	128	0.0	0.0	1	2
CNN 9 E D-25	128	128	0.25	0.25	1	2
CNN 9 E D-50	128	128	0.50	0.50	1	2

All network architectures are available as a TensorFlow 2 implementation on the dedicated Github repository^2^, together with an example of how to train the classifiers with the studied dataset.

### 4.7. Evaluation and Statistical Relevance

As main metric for comparison between models, we use the best accuracy achieved on the test set during model training. For a fair comparison to Gibas et al. (2020), we evaluate our models on subject *E*, which was used in that prior work as well.

A more detailed analysis revealed that cross-validated confusion matrices of various models are quite similar. They do not show signs of a class-specific bias, but rather exhibit a uniform distribution of misclassifications. For this reason, we omit a more detailed discussion of misclassification behavior.

Statistical relevance was evaluated using a one-way analysis of variance (ANOVA) with a significance value of *p* < 0.05. If a significant difference between the mean best accuracies was found, *post-hoc* student's *t*-tests with Holm-Bonferroni correction were performed.

## 5. Results

### 5.1. Classification Tasks

**Figure 5:**
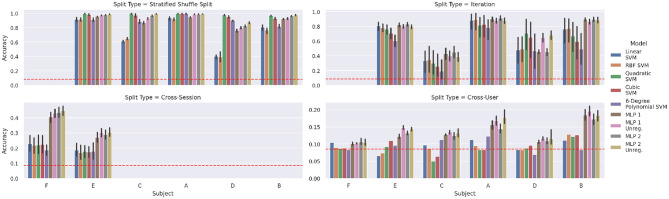
Baseline classification accuracies for verification of results from previous research using the indicated strategies for train/test splits. The dashed red line indicates the random accuracy considering a uniform distribution of samples across the 12 classes, while the vertical lines depict the respective 95% confidence intervals. To improve the statistical relevance of neural network results, each train/test setup was evaluated on five random initializations.

The results of the four classification tasks introduced in section 4.1 can be seen in the respective panels of Figure 5. The stratified shuffled split test (upper right corner) confirms that random-split in-session classification is trivial. Noticeably, in this case, even the linear kernel SVM achieves good accuracies on some subjects. We discarded data of subject *F* for this experiment, as we consider the amount of in-session data too low to yield representative results: The concerning sessions only hold a single iteration (10 samples per class) and stratified splits result in a train/test distribution of 7 vs. 3 samples per class.

Splitting by iteration, i.e., testing on one iteration and training on the others, is shown in the top right corner of Figure 5. This splitting approach has a visibly negative effect on accuracies, compared to the random-split results. The drop in accuracy is the strongest for those subjects, which were hard to handle by simple (linear) SVMs already in the random split case. This is a first indication of sensor data drift, which is studied later in section 4.3.

The cross-session experiment yields even worse results, exhibiting a drop in accuracy from 0.4 to 0.3 for MLPs and below 0.2 for SVMs. These experiments are limited to subjects *F* and *E*, because only for these we have a sufficient number of sessions available, namely 20 and 8 sessions, respectively. For both subjects, all four MLP variants do not show significant differences in performance, the same holds for the set of various SVMs. However, for subject *E*, only *MLP 1 Unreg* and *MLP 2 Unreg* yield significantly better results than the SVM models (pairwise *p* < 0.05, *d*≥1.53), whereas for subject *F* all MLPs clearly outperform the SVMs (pairwise *p* ≤ 0.001, *d*≥1.21). Overall, models trained on data of subject *F* yield a much better performance (*p* < 0.001, *d* = 0.85).

Finally, in the cross-user experiment, the MLPs are the only models that achieve a better score than a random uniform classifier for all subjects. Again, both unregularized MLPs are the only models clearly outperforming the SVMs (pairwise *p* < 0.05, *d*≥1.71).

**Figure 6:**
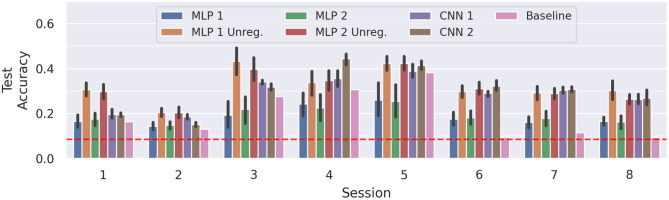
Average test accuracies of cross-session recognition on subject *E*. The vertical lines mark the 95% confidence interval and the red dotted line depicts chance level classification accuracy. The displayed data is drawn from 10 trials of evaluation performed in a leave-one-session-out cross-validation fashion, summing up to 80 training runs per model. The session number corresponding to each block denotes the session left out for testing. Note that for the baseline only one result per session is available, thus no confidence intervals can be computed.

Figure 6 compares the results of the MLPs and CNNs introduced in the present work to those introduced in Gibas et al. (2020) (denoted as *baseline*). The accuracy differences between sessions are often larger than the confidence intervals. Hence, in these configurations, the models yield rather stable learning results, but are unable to generalize well across all sessions. On average, the top performing model remains *MLP 2 Unreg* (μ = 30.45%), closely followed by *MLP 1 Unreg* (μ = 30.13%) and *MLP 2* (μ = 28.79%). Compared to the baseline scores, *MLP 2 Unreg* and *MLP 1 Unreg* increase the score significantly (*p* < 0.05, *d* = 1.44 and *p* < 0.05, *d* = 1.11, respectively). Averaged across all sessions, the results of *CNN 1* and *CNN 2*, do not differ significantly from the unregularized MLPs, and also beat the baseline significantly (*p* < 0.05 and *d*≥1.07). We also notice that our results always perform better than a random uniform classifier (red dotted line), whereas the baseline model achieved only random accuracy on sessions 6 and 8, and only slightly better on sessions 2 and 7.

**Figure 7:**
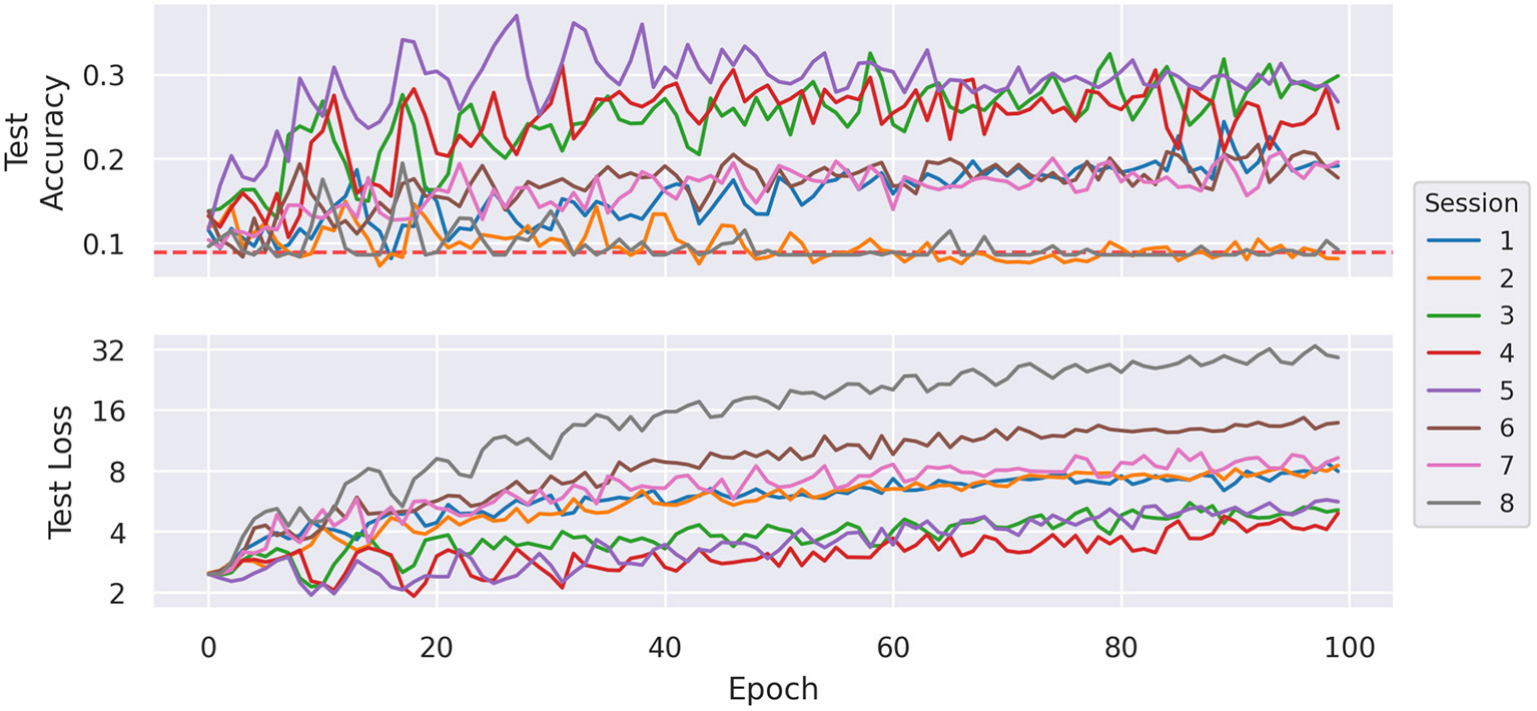
Average test accuracy and (sparse) cross-entropy loss per training epoch of *MLP 2 Unreg* by holdout session. The dashed red line indicates the accuracy of a uniform random classifier.

In Figure 7, the evolution of test accuracy and test loss are displayed for one exemplary run of each train/test configuration using *MLP 2 Unreg*. In each run, the most beneficial part of the learning process seems to be the beginning until about 20–30 epochs. In this segment, the losses of most runs roughly stay constant or even decrease, but after that, all losses tend to increase. This segment also displays the highest improvement of accuracies. The two of the weaker performing sessions, namely the runs of sessions 2 and 3, display their top accuracies in the first few epochs and then slowly converge toward random accuracy. We observe a similar behavior from runs of other models. This indicates that after this part of the training process the models overfit the training set.

### 5.2. t-SNE Analysis

**Figure 8:**
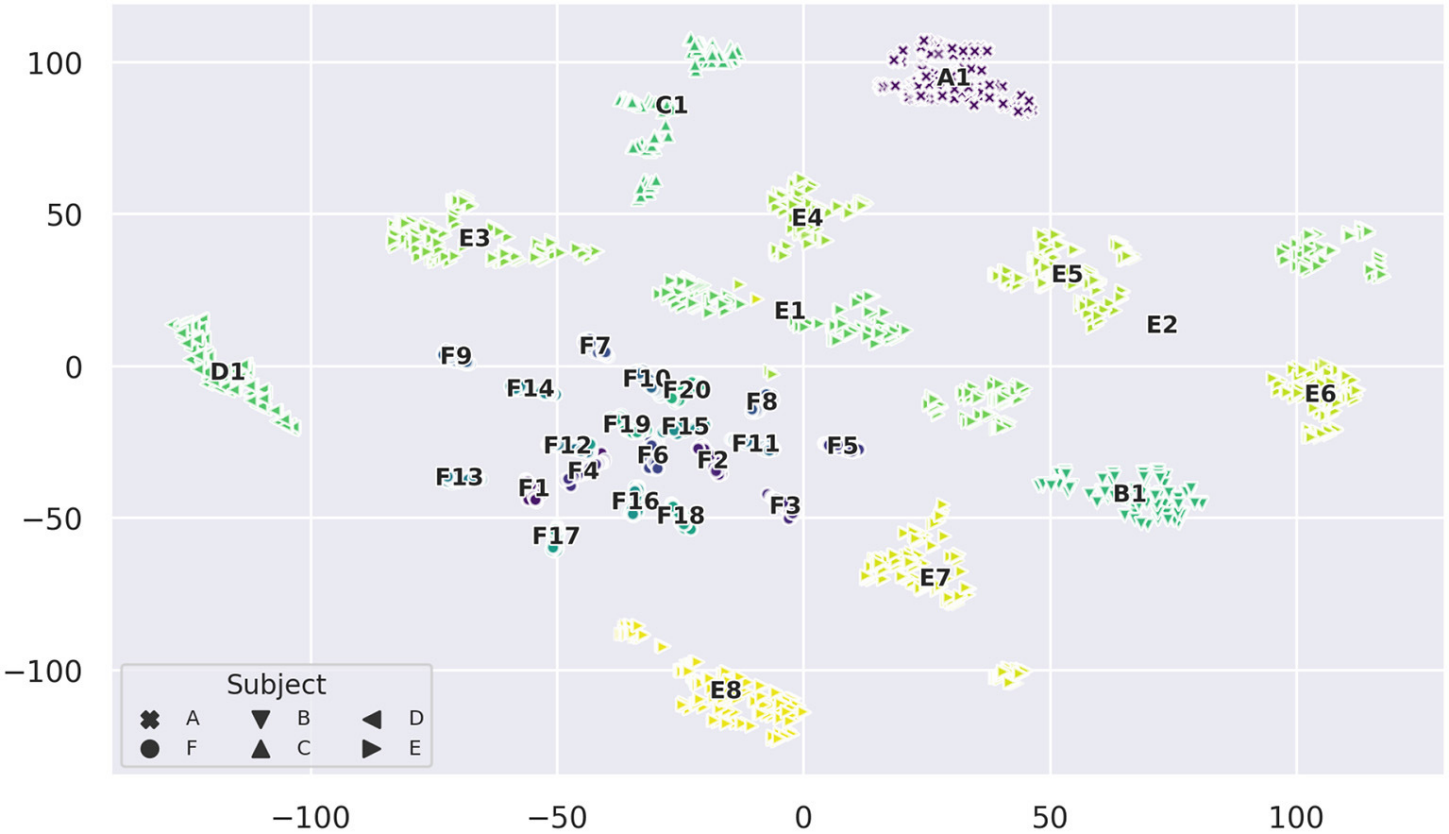
t-SNE projection of the 256-dimensional data space of raw measurements colored by session (perplexity = 90). Annotations indicate the mean sample position of a session cluster and the markers show which subject each sample comes from.

For t-SNE embeddings into a two-dimensional space, we found that clusters start to form with a perplexity of 20 or higher. These clusters resemble distinct sessions and we observed that with increasing perplexity the clusters do not visibly change in shape and distribution other than getting more compact. Figure 8 displays the almost perfect clustering of sessions that t-SNE was able to find with a perplexity of 90. A remarkable feature of this projection is that most sessions form compact clusters (except for a few outlier samples), but sessions E1, E2, E3, E7, E8, as well as C1, are either stretched or split into two or more clusters each, with session E2 having the largest distance between its two sub-clusters. However, as mentioned before, distance between clusters is not necessarily meaningful. Looking into the differences between sessions, we notice that in this embedding each session has a unique class distribution.

**Figure 9:**
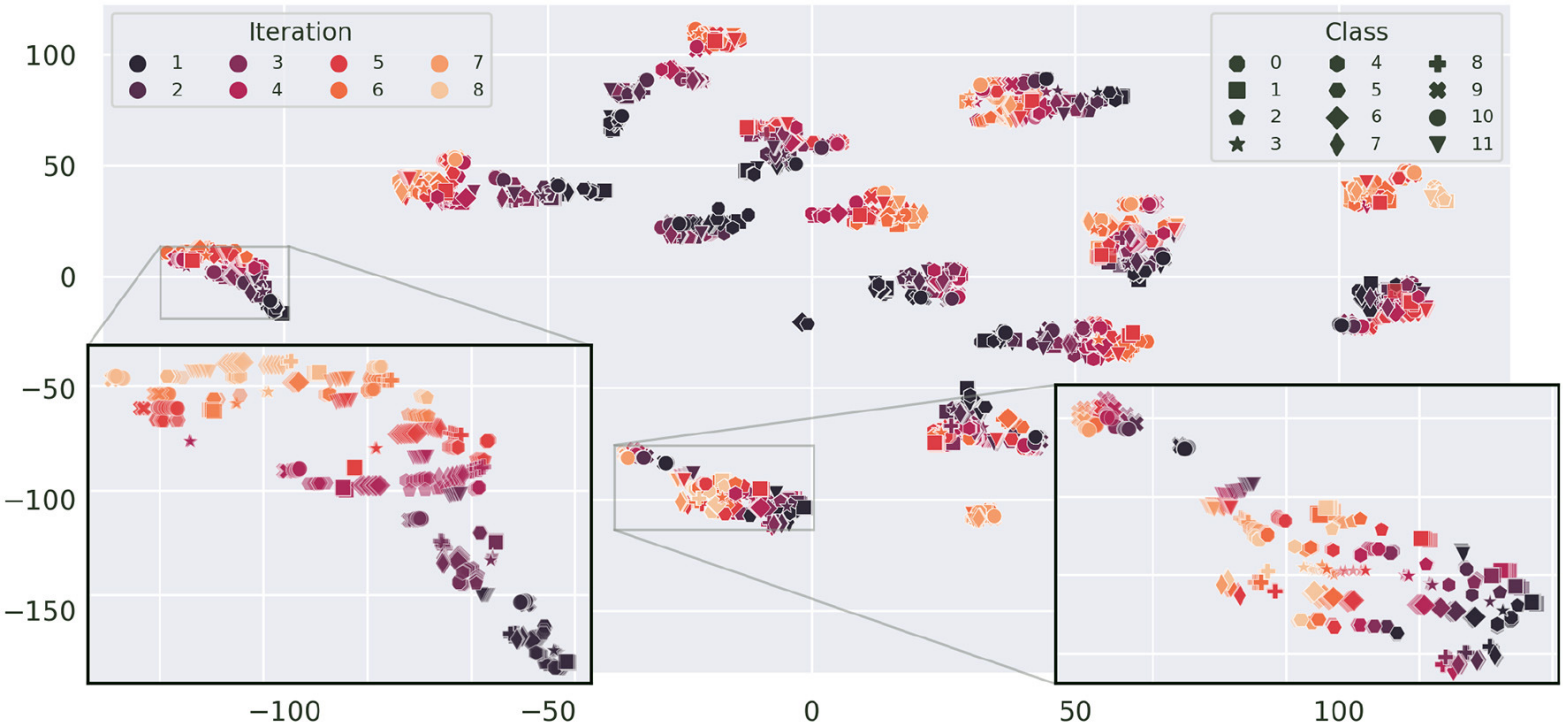
t-SNE projection of Figure 8 colorized by iteration (perplexity increased to 100). Here, classes are indicated by distinct markers. As subject *F* has a single iteration for each session only, the plot is limited to subjects A–E. The gradient from dark to light, visible in most clusters, illustrates the sample drift with increasing number of iterations. Sessions D1 **(left)** and E8 **(right)** are zoomed in upon for closer inspection.

We also observe that clusters of sessions belonging to subject *F*, which contain only a single iteration of each gesture, are much more compact. This indicates a (stronger) presence of drift in sessions with more iterations. The t-SNE projection in Figure 9 emphasizes this data drift during the course of a session by coloring the projection of Figure 8 by iteration number. In this depiction, most clusters exhibit a typical color gradient, clearly indicating a temporal drift during the course of a session. Note that some sessions are limited to 5 or 6 iterations only. Upon closer inspection we find that aforementioned sub-clusters of sessions also have a temporal correlation: For E1, iterations 1–3 and iterations 4–7 form two separate clusters. For E2 the second cluster starts after class 10 of iteration 4. E3, is stretched and displays a spatial transition starting on the right side from iteration 1 to 2 to 3, after which the remaining iterations seem to stabilize position-wise on the left. The smaller cluster belonging to E7 is composed of iteration 6 starting from class 10 and iteration 7. In C1 (from the bottom to the top), iteration 1 composes the first compact sub-cluster, iteration 2 and classes 0 and 1 of iteration 3 form the adjacent cluster, which is next to the cluster composed of iteration 4, classes 0–8, and the remaining samples form the last cluster. The close-up of the leftmost cluster (D1) gives an insightful example of the drift. The illustration does not only show a drift in terms of translation, but in later iterations the variance of the class distribution seems to increase as well. Opposed to this pattern, in E8, which is displayed in the right hand close-up, most classes seem to be separable across all iterations and later iterations only seem to introduce a little offset. Thus, in the original high-dimensional feature space, the inter-iteration variance of E8, i.e., the drift, is smaller than before.

The effect that in most cases later iterations are closer together, hints at a convergence of drift. The time between sessions and iterations was not recorded, thus no exact correlation parameters can be computed. Since E8 is the last recorded session of subject E, the very limited distortion induced by drift from the first iteration on is another hint to long-term drift convergence.

### 5.3. Cross-Iteration Drift

**Figure 10:**
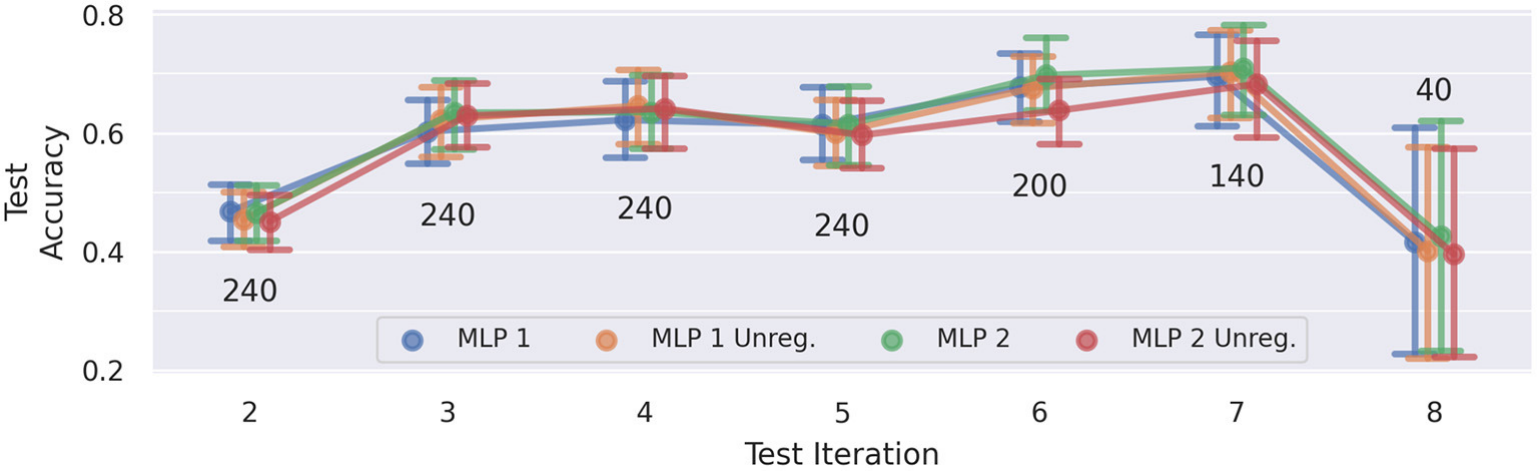
Cross-iteration prediction accuracies: a network model trained on the data of a single iteration was evaluated on the data of the subsequent iteration. Each data point of the plot represents an average across five training runs and five subjects (A–E). The number above/below a point denotes how many training runs contribute to the computed average. Error bars indicate the 95% confidence interval. Except iteration 8, accuracies increase over time, indicating a convergence of drift.

Figure 10 presents the average test results of the cross-iteration drift experiment, i.e., evaluating generalization on the named iteration, while training was performed on the previous iteration only. Again, the error bars indicate the 95% confidence intervals. The scores across all four MLP models are very similar. Since there are only two sessions that have eight iterations, the sample size for the last data point is very low, which is reflected in the rather large confidence interval for iteration 8. For this reason, we neglect iteration 8 in the following discussion of the results.

We notice a significant increase in accuracy from iteration 2 to the remaining iterations (*p* < 0.001, *d*≥0.68), and a slight improvement from iterations 3 and 5 to iteration 7 (*p* < 0.01, *d* = 0.33 and *p* < 0.01, *d* = 0.39, respectively). This could be an indication of a non-linear convergence of the drift effect.

### 5.4. Local and Global Calibration

**Figure 11:**
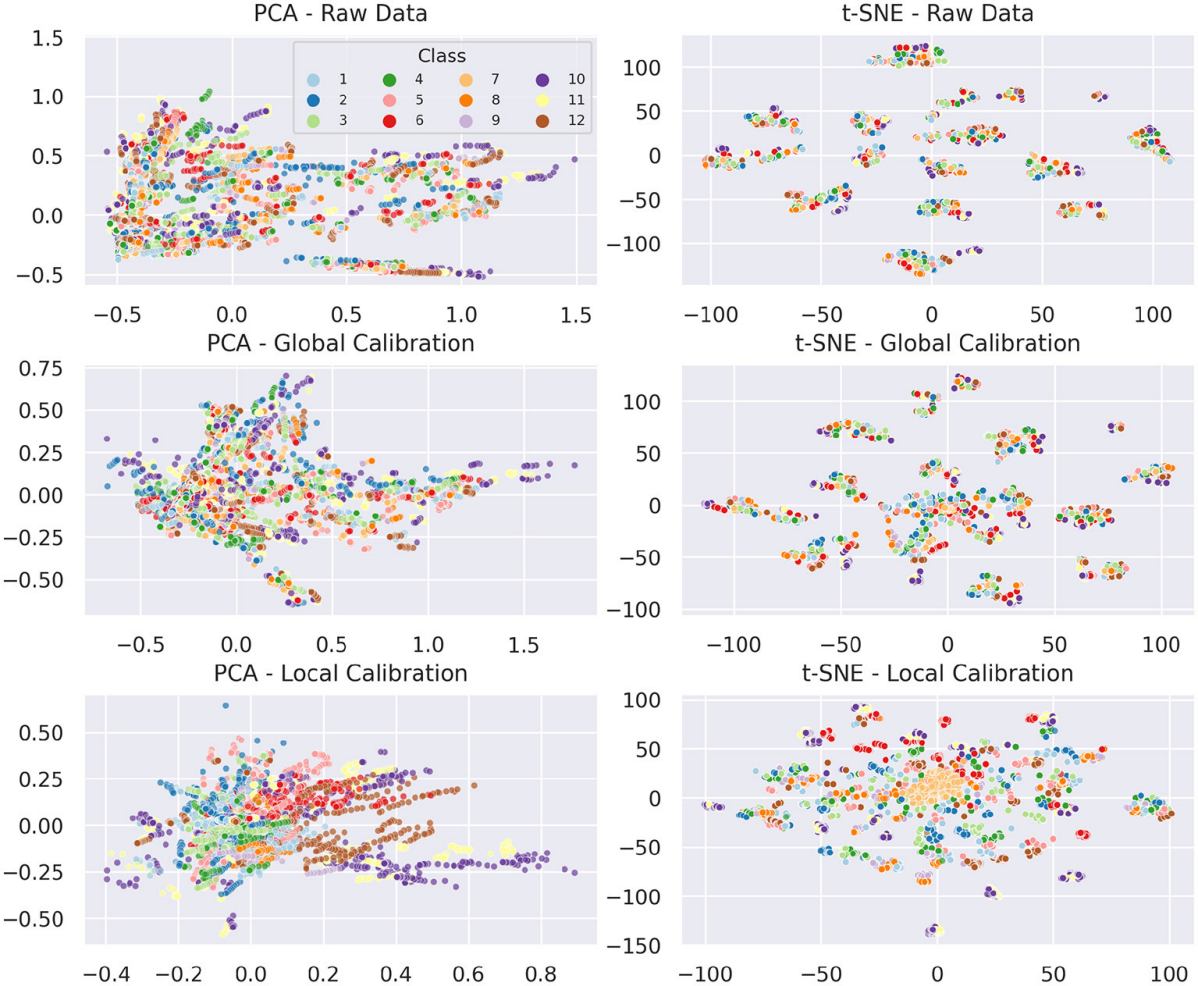
Effects of calibration on PCA and t-SNE (perplexity = 90). Data points are colored by class label. For all projections, data from subject *F* was omitted for a better comparison between local and global calibration.

To improve cross-iteration generalization and counteract drift we introduced local and global calibration in section 4.5. Figure 11 displays the effect of both types of calibrations on a PCA and a 2D t-SNE projection compared to the respective raw data projection. In the PCA projection (left column of Figure 11), the uncalibrated data forms a conical shape, mainly expanding along the x-axis. The global calibration causes the samples of a session to be centered around the origin, which corresponds to the neutral reference gesture. Consequently, the data becomes more uniform and PCA can better focus on in-session structures. The overall variance of the data is reduced. In fact, the variance of the first two principal components is reduced by 48 and 36%, respectively (from 0.224 and 0.095 to 0.117 and 0.035). The local calibration basically has the same effect on a different scale, as unification is applied per iteration instead of per session now. The variances of the first two principal components are further reduced by another 86 and 66%, respectively (from 0.117 and 0.035 to 0.016 and 0.012). The projection of the locally calibrated data already allows to better recognize groups of data points corresponding to individual classes, but that's not yet sufficient to discriminate these classes.

The t-SNE embedding of the raw data essentially resembles the characteristics already known from Figures 8, 9, namely that individual sessions form clusters and in-session drift becomes visible as a wide distribution of in-class samples across each cluster. The global calibration only modifies the formed clusters to a small extent. As mentioned before, cluster size and rotation of t-SNE projections are irrelevant and distances between clusters are not necessarily meaningful. Hence, as long as the internal structure of the clusters remains the same, they can be considered unchanged. Intuitively, the translation in the original feature space causes overlapping of the individual session distributions. This can be drawn from the fact that a new fuzzy cluster is formed roughly at the origin of the embedding space. Expectedly, all first iteration neutral samples are located at the core of the newly formed cluster. First iteration samples of several classes are dispersed loosely around this core, which should correspond to the other samples of the overlapping regions in the original feature space. Outside of the overlapping regions, distance relationships between samples remain unchanged, hence—neglecting internal structural differences caused by the absence of the samples that formed the new cluster—they form the same clusters as before. We observe the same behavior using t-SNE embeddings with much lower perplexity.

Because the local calibration causes much more overlap between sessions and iterations, the distance relationships and thus the structure of the respective t-SNE embedding is visibly changed. While all neutral samples (with exception of three data points) compose a single cluster, more and smaller clusters than before are formed. Noticeably, in almost every case for each session, each class forms a compact cluster holding samples of each iteration. This indicates that the local calibration actually achieves the intended drift mitigation.

These observations become even better visible when comparing the calibration effects isolated by session, class, and iteration. For a more in-depth study of those results, we refer to our Github repository^3^, which provides an interactive plot allowing to filter by all of these criteria.

**Figure 12:**
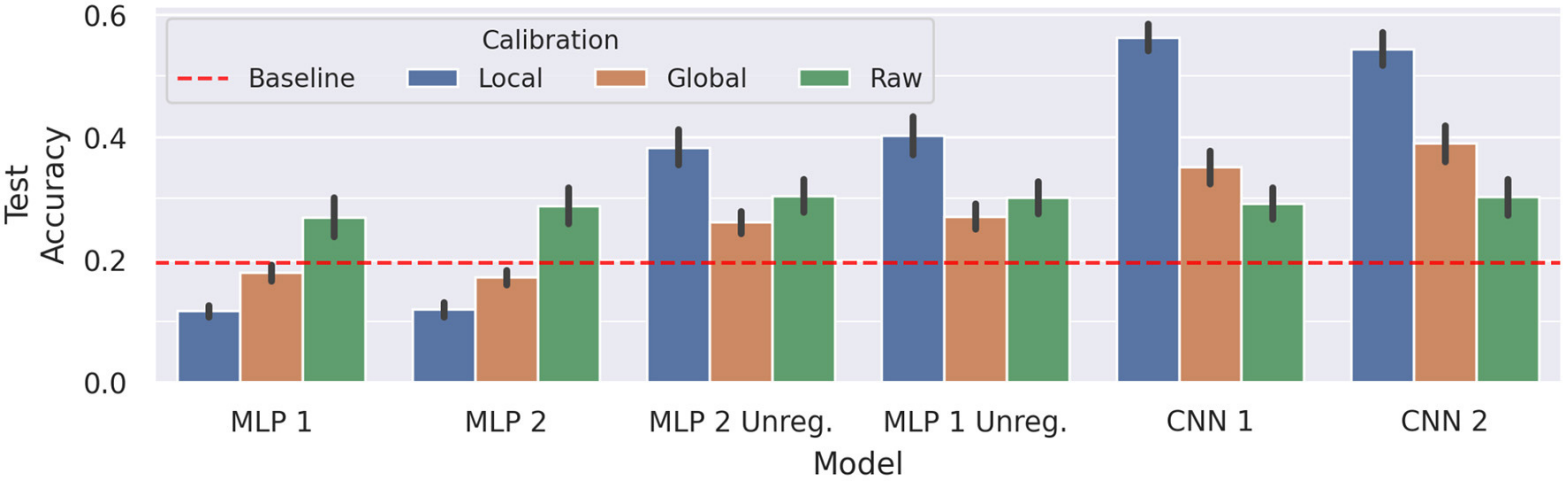
Comparison of average cross-session test accuracy on subject *E* with and without calibration. The black vertical lines display the 95% confidence intervals, whereas the red dotted line represents the average baseline score. Every train/test setup was repeated five times for each model, to account for variability due to random weight initialization.

A comparison of model performance between raw data, global calibration, and local calibration is better suited to conclude if these methods have a positive impact on classification accuracies. Results of a corresponding analysis are shown in Figure 12. Both *MLP 1* and *MLP 2* yield slightly worse results for global calibration (*p* < 0.001, *d* = 1.14, and *p* < 0.001, *d* = 1.56) and much worse results for local calibration (*p* < 0.001, *d* = 2.02 and *p* < 0.001, *d* = 2.29). On the other hand, while for both unregularized models global calibration causes a slight decrease in test accuracy (insignificant for *MLP 1 Unreg* and *p* < 0.05, *d* = 0.57 for *MLP 2 Unreg*), local calibration achieves visibly better results (*p* < 0.001, *d* = 1.08 and *p* < 0.001, *d* = 0.89).

The same procedure of testing calibration effectivity was repeated for the CNNs introduced in section 4.2. While the raw data results of the CNNs do not differ much from the ones of the previous models, they can evidently better leverage the calibrated data: For both *CNN 1* and *CNN 2* global calibration results in an average increase of about 6% for *CNN 1* (*p* < 0.01, *d* = 0.7) to a total of 35.12%, and about 9% for *CNN 2* (*p* < 0.001, *d* = 0.91) to a total of 39%. The local calibration raises performance even higher to 56.34% for *CNN 1* (*p* < 0.001, *d* = 3.51) and to 54.36% for *CNN 2* (*p* < 0.001, *d* = 2.64).

### 5.5. Learned Calibration

**Figure 13:**
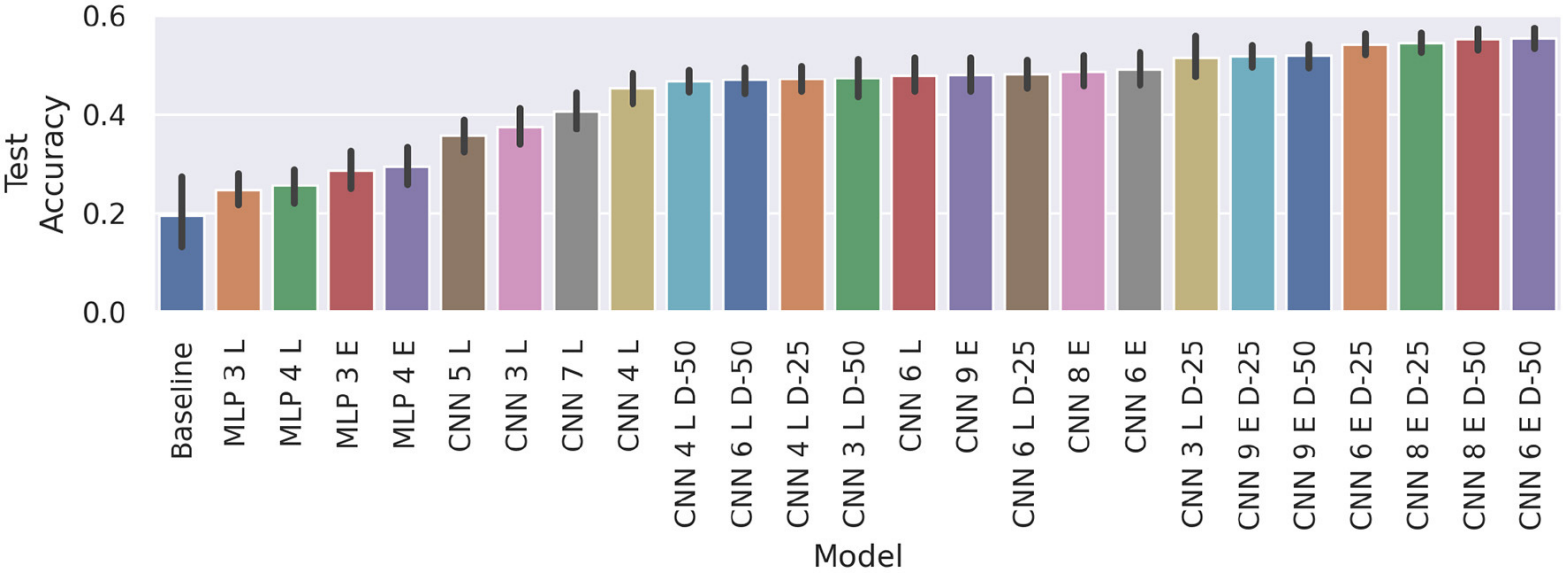
Comparison of average cross-session test accuracy on subject *E* with learned calibration and our baseline. The black vertical lines display the 95% confidence intervals. Again, every train/test setup was trained five times on each model, to achieve statistical robustness. Suffixes “E” and “L” denote early and late fusion architectures, respectively.

To further increase the effectiveness of calibration, we enabled the networks themselves to find a suitable calibration method, providing the averaged neutral pose of the considered iteration as a second input to the network (cf. section 4.6). The corresponding results are shown in Figure 13. The MLP variants are not able to draw useful information from the additional data, they even perform slightly but insignificantly worse than previous MLP models trained on raw data only.

All CNNs, on the other hand, clearly beat the baseline (*CNN 5 L* and *CNN 3 L* with *p* < .05, *d*≥1.53, *CNN 7 L* with *p* < 0.01, *d* = 1.88, and the remaining ones with *p* < 0.001, *d*≥2.52). The models *CNN 4 L D-50* through *CNN 6 E D-50* also manage to beat the global calibration scores of *CNN 1* (*p* < 0.001, *d*≥1.11) and *CNN 2* (*p* < 0.05, *d*≥0.72).

Unlike before, where unregularized models performed better, adding dropout to dual input models either improves the result or does not change the outcome significantly. For example, *CNN 4 L, CNN 6 L*, and *CNN 9 E*, using dropout does not make a big difference. On the other hand, adding dropout layers with 25 or 50% dropout rate after the convolutions, improves the performance of *CNN 3 L* (*p* < 0.001, *d*≥0.81), *CNN 6 E* (*p* < 0.05, *d* = 0.49, and *p* < 0.01, *d* = 0.61), and *CNN 8 E* (*p* < 0.01, *d*≥0.59). This indicates that the early fusion models generally benefit more from dropout regularization. In our results, we identify a clear benefit of early fusion over late fusion models (*p* < 0.001, *d* = 0.89). We also notice a saturation of average best test accuracy at about 50–55%.

**Figure 14:**
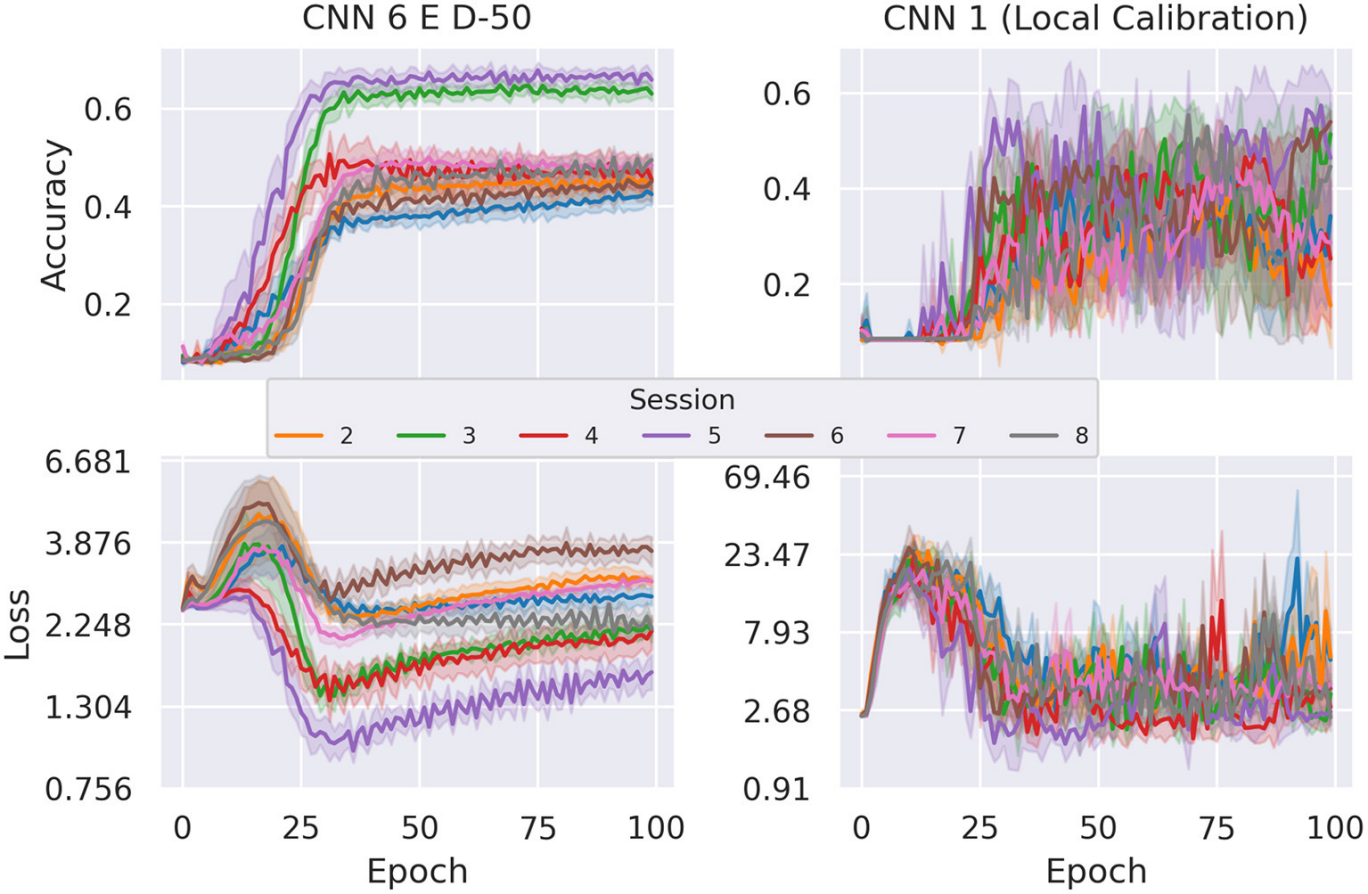
Comparison of average cross-session test accuracy and loss on holdout sessions of subject *E* between dual input and local calibration single input network. This depiction clearly shows that the dual input model's test accuracy and loss are more stable. The error bands depict the 95% confidence intervals.

*CNN 6 E D-50*—the best performing learned-calibration model—with an average classification rate of 55.37%, reaches about the same performance level achieved with local calibration (with insignificant deviation). However, a comparison of the evolutions of test loss and test accuracy over time reveals that the dual input networks exhibit much more robust training. This can be observed in Figure 14, where we compare our best performing dual-input model (*CNN 6 E D-50*) with our best performing standard model (*CNN 1*) running on locally calibrated data. Stability in this case not only refers to the lower fluctuation of average values between epochs, but also to the fact that different random initializations of the model achieve more similar results. This can be concluded from the more narrow error bands indicating 95% confidence intervals. Furthermore, the loss of the dual-input model is also noticeably smaller than that of the manually calibrated model.

Analogously to the single input global calibration, we also evaluated a calibration approach that only considers the 10 neutral pose measurements of the very first iteration and employing this single reference for all other iterations. However, the training results on all considered network architectures only achieved random-level test accuracies, again emphasizing the importance of iteration-local calibration to handle sensor drift.

## 6. Discussion

The lack of standards in this relatively young field of research, e.g., considering the number and type of gestures used or the large variety of custom-made EIT devices and measuring techniques employed, limit the comparability of results and allow only general conclusions. With regard to this, our initial classification results reported in section 5.1 are mostly consistent with the findings of the previous works, with exception of the much better cross-session and cross-user accuracies reported by Zhang and Harrison (2015).

Based on this preliminary evaluation, we choose to mainly focus on the further improvement of cross-session prediction results. Because the studied dataset was originally recorded for Gibas et al. (2020) (except subject *F*), we used their cross-session results as a baseline to measure increase in performance.

The fact that on average subject *F* yields a much better performance suggests that more training data—as usual—increases the generalization capabilities of the trained models. Another influencing factor is that the data of subject *F* is less distorted by drift, as only a single iteration was recorded for each gesture. Still, the best accuracies achieved with the MLPs are not yet suitable for an actual application. An interesting question for further research would be to find the number of sessions that makes the positive effect of data variance saturate. However, this is bound to the specific device and the study design.

While a network with 2 hidden layers might already be considered a deep neural network, the architectures we employed so far are very basic, as the small number of layers limits the networks' capability of modeling high-level features as a composition of simpler ones. With that in mind, the best classification result of 50.23% accuracy (achieved by *MLP 1* on session 5) on raw data is a promising result that gives hope for even better cross-session results when trained on more sophisticated network architectures and/or more data. Figure 7 demonstrates that the first 20–25 epochs have the highest impact on the test accuracy and later epochs cause the accuracy to jitter. The same effect seems to be even more prominent for local and learned calibration, as can be observed in Figure 14. Looking only at the accuracies this behavior could be explained by an inappropriate learning rate preventing the model to converge to its optimum. But several tests with varying batch sizes, learning rates, and training epochs displayed similar behavior differently stretched throughout the training process. Thus, we rule out an incorrectly chosen learning rate and attribute this to overfitting of the training set.

The t-SNE projections give an impression of how data is distributed in the original feature space, which allows for some conclusions about the data domain and inferring reasons for the low classification accuracies the models achieved. The results of these analyses confirm that the in-session similarity of data points is higher than the cross-session class similarity, which is the core reason why cross-session classification becomes so difficult.

The t-SNE embedding reveals that even sessions of the same subject form distinct clusters with strongly different local geometries, which correspond to different spatial distributions of classes in the original feature space. Thus, physiological differences can be ruled out as a cause for the unique distributions. We conclude that even slight differences in the placement of the EIT device, and (inherently) differing contact impedances result in a significant change of the sample distribution. Based on these observations we conclude that cross-session learning poses the problem of anticipating the local geometry of an unseen cluster, i.e., the session-specific spatial class distribution (including its drift), which is expected to strongly vary from already seen examples.

The iteration drift experiment was carried out to gain some insight into how the drift affects the generalization capability to the next iteration. The results do not allow a conclusive assessment, but we hypothesize a convergence of drift. This could mean that the drift is mainly rooted in the changing measurement magnitudes due to heat development of the electrodes until the operating temperature is reached. The degradation effect is much stronger when the first iteration is used for training and the second for evaluation, and then strongly decreases in later iterations. This could be explained by a non-linear heating process of the electrodes, where the initial strong effect is caused by a cold start and later convergence is explained by the temperature converging over time.

Unfortunately, we do not have enough data available to further narrow down the exact characteristics and cause of the drift. However, the problems of drift and gesture recognition can be tackled separately. Thus, we came up with the calibration approaches to further analyze the classification problem without fully understanding the drift yet.

The projections comparing raw data with calibrated data did show the effects we wanted to achieve, however, in the ideal case, an embedding would display well-defined clusters for each class, but this is not the case for either of the calibrations. On the other hand, it is important to note that here we mainly used t-SNE and PCA for the purpose of calibration effect visualization. Though these projections try to preserve as much information as possible, the reduction from 256 to 2 feature dimensions entails a significant loss of information. Hence, the projections alone can not be used to determine if the calibrations are advantageous or not.

The performance of the previously used models on the calibrated data surprisingly shows that the MLPs regularized by dropout yield worse results in both cases, whereas the unregularized variants only perform better with locally calibrated data. In contrast to that, the CNNs are much better at leveraging the calibration effects, but do not provide any benefit on the raw data.

The learned calibration results show that in this case the ability to leverage spatial information also proves to be advantageous: While the MLPs, regardless of employing single or dual input, do not yield better results than the previous raw data results, the CNNs manage to get similar or even better results than the previous global calibration experiments. But they do not manage to beat the best local calibration results.

Even if the best learned-calibration models only reach the same level of accuracy of local calibration results, the direct comparison of the accuracy and loss evolutions during training shows that learned calibration should be preferred. It becomes apparent that the higher average scores of the single input models are due to the high variance in accuracy. However, the greater stability of the dual-input models is a more desirable property for the models, because then they are not only less affected by random initialization, but also after 25–30 training epochs the test accuracy is far less dependent on when the training is stopped.

## 7. Conclusion and Outlook

In our analysis, we were able to confirm that in-session gesture recognition can be considered rather trivial, but that cross-session recognition poses a challenging research problem because the data distribution between sessions varies much more than between classes, even if the data was recorded from the same subject. This variance can be attributed to static differences in contact impedances rooted in slightly different placements of the EIT device, as well as dynamic impedance changes, e.g., caused by changes in electrode temperatures. We presented different calibration techniques that improve classification results on offline data up to an average of 56%, which beats previous results on the same data by 37%. The dual input models receiving the average neutral pose of each iteration as secondary input achieve promising results, as they are not only less sensitive to random weight initialization than the previous models, but the optimization steps also seem to have a more predictable effect on the test data. However, these results require a local calibration, employing the neutral gesture of each individual iteration for calibration. An approach that only employs the calibration gesture of the first iteration does not achieve acceptable results yet. We believe this is due to the strong drift we found to be present in the data and without contextual information we derive from the study setup our models are unable to overcome the difficulty imposed by the drift. Because of this, we think it will be necessary to better understand and model the properties of drift to achieve acceptable classification rates with online data in a natural scenario. Therefore, a thorough analysis of drift will play an important role in our further research.

Up to this point, we focused on very basic architectures with a small number of layers. Without question, these models leave much room for further improvement toward specialization for the specific data domain. However, a highly specialized model requires a more thorough exploration of the layer architecture and its hyper-parameters and without established best practices for this specific data domain, it may take a while until we are able to achieve as good results with neural networks as we are used to from other research fields. We also notice that these basic architectures tend to fit the train set quite good after only a few training epochs, while the test losses tend to increase over time. Another interesting property of the trained models is that misclassifications across all gestures are rather uniformly distributed. This behavior suggests that the models struggle with generalization across high session variability, while maintaining the high class separability that we observe within sessions. This is an indication that the problem does not lie in gestures being too indistinguishable, and can eventually be solved with more data and more sophisticated network architectures.

We did also consider data augmentation techniques to generate additional training data, but currently these methods are limited to a circular shifting of the cycles in a frame, thus simulating a rotation of the device by 360°/16 such that electrodes just switch places. However, this will only achieve invariance for this discrete type of rotation, which is equivalent to arbitrarily choosing the electrode pair where current is injected in the first cycle of a frame. With a resolution of 16 electrodes, we consider this augmentation not to be very useful and corresponding experiments confirmed this concern, i.e., did not improve the test set accuracy significantly. Yet, at our current state of knowledge, we are limited to this augmentation method, because we are unable to verify if newly generated data is close to reality. Further experiments with phantoms could prove useful to find augmentation patterns regarding drift and rotation that also apply to measurements on a human forearm. Notably, a deeper understanding of the data distribution and the underlying data would also allow the application of generative models such as generative adversarial nets (GANs) (Goodfellow et al., 2014) and variational autoencoders (VAEs) (Kingma and Welling, 2013), which try to learn the underlying generative factors of the available data and are not only able to generate new samples, but can also be leveraged for classification purposes.

## Data Availability Statement

The datasets presented in this study can be found in online repositories. The names of the repository/repositories and accession number(s) can be found at: https://doi.org/10.4119/unibi/2948441.

## Ethics Statement

Ethical review and approval was not required for the study on human participants in accordance with the local legislation and institutional requirements. The patients/participants provided their written informed consent to participate in this study. Written informed consent was obtained from the individual(s) for the publication of any potentially identifiable images or data included in this article.

## Author Contributions

The EIT system used in this work was developed by CG and RB. Data was also recorded by CG and RB. DL conducted the data analysis, designed the new network architectures, and wrote the code. The results were interpreted by DL and RH. All authors contributed to manuscript revision, read, and approved the submitted version.

## Conflict of Interest

The authors declare that the research was conducted in the absence of any commercial or financial relationships that could be construed as a potential conflict of interest.

## Publisher's Note

All claims expressed in this article are solely those of the authors and do not necessarily represent those of their affiliated organizations, or those of the publisher, the editors and the reviewers. Any product that may be evaluated in this article, or claim that may be made by its manufacturer, is not guaranteed or endorsed by the publisher.

## Footnotes

^1^ https://www.mobiusbionics.com

^2^https://github.com/DavidPL1/eit_data_analysis.

^3^https://github.com/DavidPL1/eit_data_analysis.
